# Characterization of the Influenza A H5N1 Viruses of the 2008-09 Outbreaks in India Reveals a Third Introduction and Possible Endemicity

**DOI:** 10.1371/journal.pone.0007846

**Published:** 2009-11-16

**Authors:** Alok K. Chakrabarti, Shailesh D. Pawar, Sarah S. Cherian, Santosh S. Koratkar, Santosh M. Jadhav, Biswajoy Pal, Satish Raut, Vishal Thite, Sadhana S. Kode, Sachin S. Keng, Bestin J. Payyapilly, Jayati Mullick, Akhilesh C. Mishra

**Affiliations:** Microbial Containment Complex, National Institute of Virology, Pune, India; Institut Pasteur, France

## Abstract

Widespread infection of highly pathogenic avian influenza A H5N1 was reported from backyard and commercial poultry in West Bengal (WB), an eastern state of India in early 2008. Infection gradually spread to Tripura, Assam and Sikkim, the northeastern states, with 70 outbreaks reported between January 2008 and May 2009. Whole genome sequence analysis of three isolates from WB, one isolate from Tripura along with the analysis of hemagglutinin (HA) and neuraminidase (NA) genes of 17 other isolates was performed during this study. In the HA gene phylogenetic tree, all the 2008-09 Indian isolates belonged to EMA3 sublineage of clade 2.2. The closest phylogenetic relationship was found to be with the 2007-09 isolates from Bangladesh and not with the earlier 2006 and 2007 Indian isolates implying a third introduction into the country. The receptor-binding pocket of HA1 of two isolates from WB showed S221P mutation, one of the markers predicted to be associated with human receptor specificity. Two substitutions E119A (2 isolates of WB) and N294S (2 other isolates of WB) known to confer resistance to NA inhibitors were observed in the active site of neuraminidase. Several additional mutations were observed within the 2008-09 Indian isolates indicating genetic diversification. Overall, the study is indicative of a possible endemicity in the eastern and northeastern parts of the country, demanding active surveillance specifically in view of the critical mutations that have been observed in the influenza A H5N1 viruses.

## Introduction

Highly pathogenic avian influenza (HPAI) A H5N1 viruses continue to pose a serious threat to global public health. As of May 2009, 424 confirmed human cases resulting in 261 deaths have been reported from 15 countries [1]. Evolution and divergence of H5N1 viruses continues and isolates from Europe, Africa and the Middle East are classified into clade 2.2, Qinghai-like viruses [2]. Several recent reports [3]–[6] describe the further evolution of clade 2.2 viruses and identify emerging sublineages. The EMA 1–3 sublineages [6] represent the viruses isolated since 2005 from Europe, Middle East and Africa as well as isolates from China, Russia, and Mongolia. The EMA-1 sublineage includes amongst others, isolates from Czech Republic, Turkey, Egypt, Nigeria, Mongolia, Kurgan and Novosibirsk. The EMA-2 includes isolates from Denmark, Scotland, Germany, Nigeria, Astrakhan and Krasnoozerka while EMA-3 includes isolates from Afghanistan, Mongolia, Italy, Iran and Krasnodar. A minority of isolates belonging to Qinghai, Novosibirsk region, Shantou and Omsk did not group with either of these sublineages and have been left unassigned.

India experienced the first outbreak of HPAI H5N1 in domestic poultry from January 2006 through April 2006 [7] in parts of the western states Maharashtra and Gujarat and a central state Madhya Pradesh. Genomic characterization [8] revealed that the virus belonged to the clade 2.2, EMA-3 sublineage [6]. Control measures adopted helped combating the virus and declaring the country free of the virus in August 2006 [7]. The second outbreak was reported from backyard poultry in Manipur, a northeast state in July 2007 [9]. The virus was characterized as a unique one, distinctly different from the viruses of the EMA sublineages and considered to have been an independent introduction into the country. During 2008, wide spread infection of influenza A H5N1 was reported in backyard and commercial poultry in (WB), an eastern state of India and later in Tripura, a north-eastern state. A total of 39 outbreaks were reported in WB and 3 outbreaks in Tripura in the phase I of the infection during January to May 2008 [10]. After successful control and containment operations, the country was declared free of the virus on 4^th^ November 2008. However, a second phase of the disease was reported from 27^th^ November 2008 to May 2009 in the northeastern state of Assam (18 outbreaks), Sikkim (1 outbreak) and WB (9 outbreaks) [11]. Overall, during the period from January 2008 to May 2009, 70 outbreaks of the H5N1 infection occurred causing 131,614 (0.13 million) poultry deaths and involving the culling of about 10.5 million poultry [10], [11].

The aim of the present study was to describe the recent outbreaks and genetically characterize the Indian isolates of WB, Assam and Tripura to understand the genetic diversity and significant mutations.

## Results

**Figure 1:**
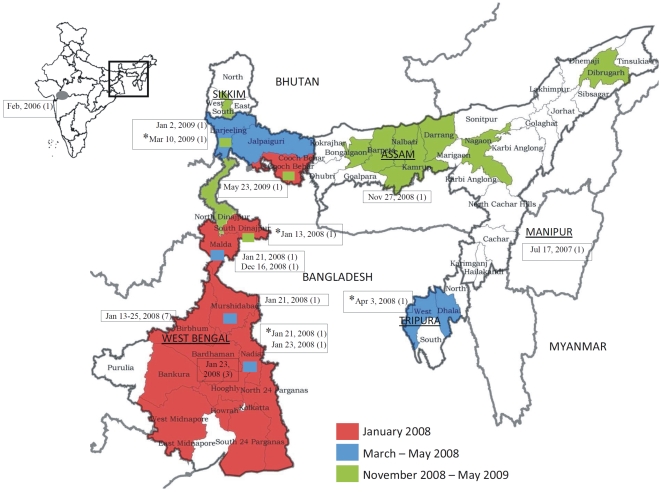
Location of highly pathogenic avian influenza (HPAI) Influenza A H5N1 virus outbreaks during 2008-09 in India. Indian states shown as underlined uppercase, districts in lowercase and neighbouring countries in uppercase font. The phase wise spread is as indicated in the colour scheme. Inset shows the earliest Indian H5N1 outbreak of 2006 (grey oval). Black box in the inset shows the area of enlargement. Rectangular boxes attached to different locations in the map denote the place of isolation and include the isolation dates along with the number in the parenthesis denoting the number of isolates. * indicates the isolates whose full genome has been sequenced.

Seventeen of 18 districts in WB, eight of 27 districts in Assam, two of 4 districts in Tripura and South Sikkim were affected during the two phases of the HPAI H5N1 infection from January 2008 to May 2009. As illustrated in Figure 1, 15 districts of WB were affected in the first month of phase I (shown in red) which further spread to additional districts of WB and Tripura and reoccured in some of the previously affected districts of WB by the end of the phase I (shown in blue). After the country was declared free of H5N1 on November 4^th^, 2008, there was another occurrence of H5N1 termed as phase II in the states depicted in green (Figure 1).

### Isolation and Identification of Avian Influenza Viruses

In the present study, nineteen isolates from WB and one each from the states of Tripura and Assam were obtained. Whole genome sequencing was performed for four isolates including three from WB (Ck/India/WB-NIV529/08, Ck/India/WB-NIV2653/08 and Ck/India/WB-2456/09) and one from Tripura (Ck/India/TR-NIV4396/08). In addition, sixteen other HA and NA genes from WB isolates and one HA and NA gene from the Assam isolates were also sequenced. GenBank accession numbers for the gene segments of the WB and Tripura isolates of January 2008–April 2008 are from CY046067 to CY046116 and the accession numbers for isolates from Assam and WB, December 2008–May 2009 are from GQ917223 to GQ917238.

### Phylogenetic Analysis

**Figure 2:**
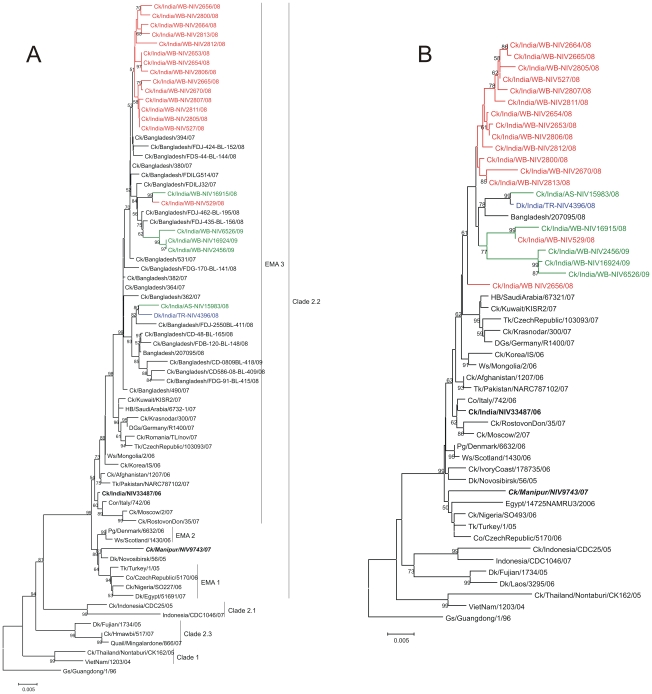
The HA (A) and NA (B) phylogenetic trees constructed by using the neighbour-joining method as implemented in MEGA. Scale bar indicates number of nucleotide substitutions per site. Gs/Guangdong/1/96 was used as out group sequence. Gs/Guangdong/1/96 was used as out group sequence. Abbreviations: Ck-Chicken, Dk-Duck, Gs-Goose, Ws-Whooper swan, Tk-Turkey, Pg-Peregrine, HB-Houbara Bustard, CO-Cygnus Olor, DGs-Domestic Goose.

In the hemagglutinin (HA) phylogenetic tree (Figure 2A), the WB, Tripura and Assam isolates (henceforth referred to as the 2008-09 Indian isolates) clustered together with 100% bootstrap support. Notably, the cluster also consisted of Bangladesh sequences of the period from 2007–2009. The combined cluster of Indian and Bangladesh isolates were closely related to the 2007 isolates from Kuwait, Saudi Arabia, Germany, Krasnodar and the whole group was further close to isolates from Mongolia, Afghanistan, Pakistan and India 2006 (Figure 2A). All these belong to EMA-3 sublineage of clade 2.2 viruses. Amongst the Indian and Bangladesh sequences, the isolate Ck/Bangladesh/364/07 showed maximum identity with a Saudi Arabia isolate Hb/SA/67321/07 and Kuwait isolate Ck/KT/KISR2/07. The percent nucleotide identity (PNI) and percent amino acid identity (PAI) in HA gene with Hb/SA/67321/07 isolate was 99.45 and 99.64 while with Ck/KT/KISR2/07 was 99.39 and 99.82, respectively. Among the 2008-09 Indian isolates, Ck/India/WB-NIV2805/08 showed maximum identity, PNI 99.03 and PAI 99.46 with Hb/SA/67321/07 while PNI 98.97 and PAI 99.64 with Ck/KT/KISR2/07. Between isolates Ck/Bangladesh/364/07 and Ck/India/WB-NIV2805/08 the PNI was 99.58 and the PAI was 99.82.

The overall similarity of the 2008-09 Indian isolates with the 2007-09 Bangladesh isolates was 98.90±0.12 (PNI) and 99.10±0.19 (PAI) while the similarity of the 2008-09 Indian isolates with Ck/Bangladesh/364/07 was 99.23±0.12 (PNI) and 99.41±0.15 (PAI) The PNI and PAI values of 2008-09 Indian isolates with the 2006 Indian isolate [8] were 98.33±0.26 and 98.88±0.34, respectively while with the 2007 isolate [9] were 96.99±0.37 and 98.05±0.50, respectively. Considering the closeness of the 2008-09 Indian isolates with the Bangladesh 2007 isolates and not with the earlier Indian isolates of 2006 and 2007 may imply a re-introduction of the virus into the country.

**Figure 3:**
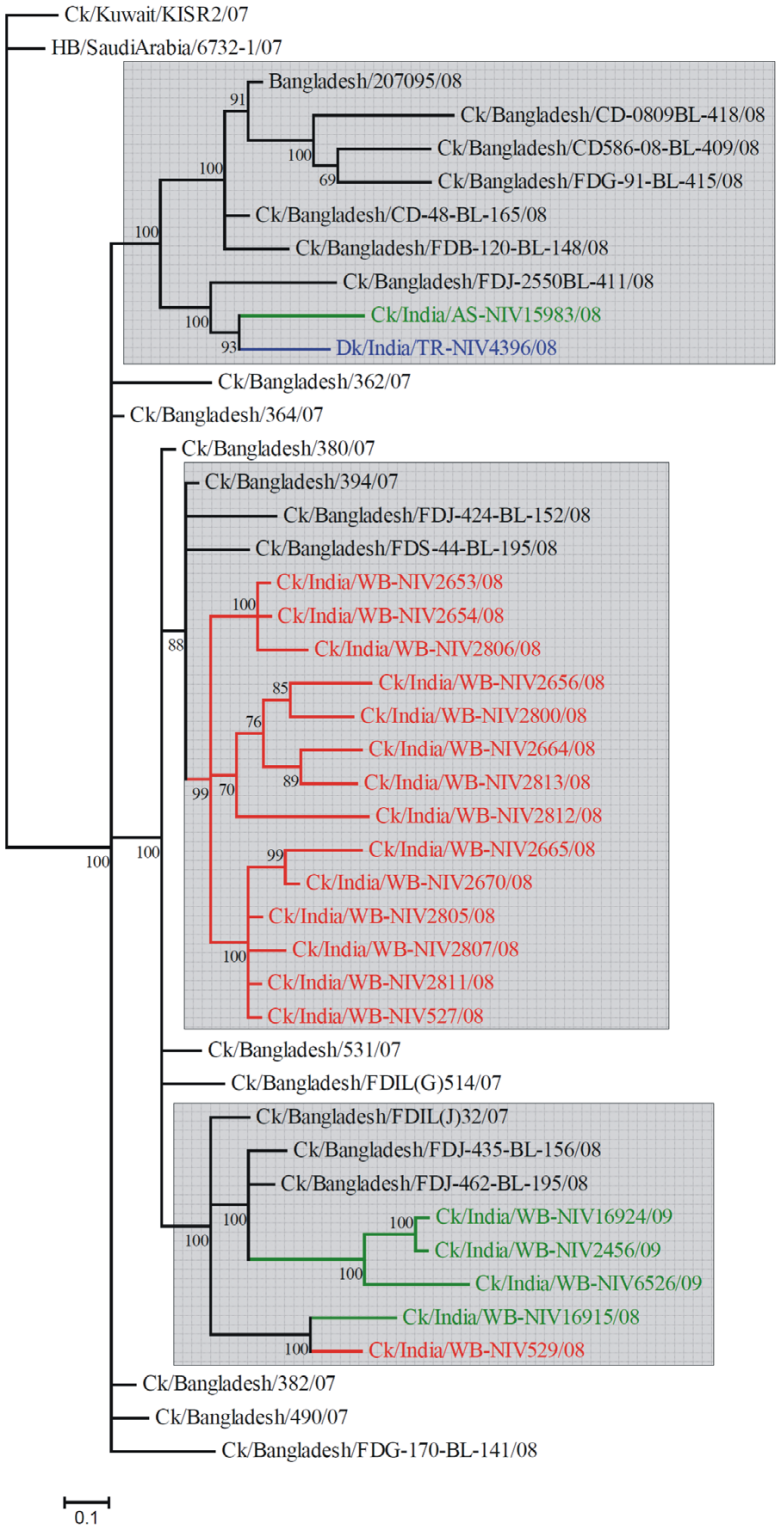
HA gene phylogeny of the 2008-09 Indian isolates and 2007-09 Bangladesh isolates using the Bayesian method.

Overall, the divergence within the 2008-09 Indian isolates was 1.04% at nucleotide level and 0.93% at amino acid level. Within the cluster of Indian and Bangladesh isolates (Figure 3), three main subgroups with very high bootstrap support were observed. In each of these subgroups, the Indian isolates clustered together with some of the isolates from Bangladesh. One group consisted of the early Indian isolates of January 2008 from southern part of WB (Figure 1), which formed a close group (PNI within group being 99.46 and PAI 99.48) having 99% bootstrap support (Figure 3). The Bangladesh isolates (Ck/Bangladesh/394/07, Ck/Bangladesh/FDJ-424-BL-152/08 and Ck/Bangladesh/FDS-44-BL195/08) fell outside this cluster of the early 2008 Indian isolates. In the next group an Indian isolate of early 2008 (Ck/India/WB-NIV529/08) and another Indian isolate of December 2008 (Ck/India/WB-NIV16915/08) showed relatedness with a Bangladesh isolate Ck/Bangladesh/FDILJ32/07 and another distinct cluster (PNI within the group being 99.60 and PAI 99.88) which consisted of three Indian isolates of 2009 (corresponding to phase II of the Indian outbreaks). Incidentally, two isolates from Bangladesh, Ck/Bangladesh/FDJ-435-BL-156/08 and Ck/Bangladesh/FDJ-462-BL-195/08 showed relatedness with the phase II Indian isolates. In the third group, the Indian isolates of Tripura (Dk/India/TR-NIV4396/08) and Assam (Ck/India/AS-NIV/15983/08) grouped together with an isolate Ck/Bangladesh/FDJ-2550BL-411/08 of Bangladesh. A human isolate of Bangladesh (A/Bangladesh/207095/08) [12] clustered separately in this group with five other Bangladesh isolates. In the NA gene, the overall topology of the phylogenetic tree (Figure 2B) was the same as that in HA (Figure 2A). The human isolate from Bangladesh fell in the group of isolates from Tripura and Assam. The divergence within the 2008-09 Indian isolates was 1.15% at the nucleotide level and 1.59% at the amino acid level. The PNI (PAI) of the Indian 2008-09 isolates with the closest isolate from Kuwait was 98.68±0.25 (98.66±0.33) while with the Saudi Arabia isolate was 98.68±0.24 (98.46±0.39). The Indian isolates shared closest identity with the Bangladesh isolate (A/Bangladesh/207095/08) with PNI 98.73±0.24 and PAI 98.81±0.30. The PNI and PAI with respect to the India 2006 isolate was 98.66±0.24 and 98.28±0.43, while with India 2007 isolate was 97.54±0.35 and 96.73±0.70, respectively. The phylogenetic tree for all the other genes (data not shown) also showed similar overall topology as the HA and NA trees indicating that none of the isolates studied here were reassortants.

Several mutations were observed within the 2008-09 Indian isolates indicating genetic diversity (Table 1, 2). A substitution A496S in HA (H5 numbering and reference as Ck/India/NIV33487/06) was found in all 2008-09 Indian isolates as well as all 2007-09 Bangladesh isolates as in the 2007 isolates of Kuwait, Saudi Arabia, Krasnodar and Germany/R1400/07. Another substitution (R162I was found in all 2008-09 Indian isolates (except Dk/India/TR-NIV4396/08) and majority of 2007-09 Bangladesh isolates as in the 2007 isolates of Kuwait, Saudi Arabia, Krasnodar and Germany/R1400/07. A substitution Q207L was uniquely shared between all 2008-09 Indian isolates and Bangladesh isolates (except Ck/Bangladesh/FDJ-424-BL-152/08). The early 2008 Indian isolates (except Ck/India/WB-NIV529/08) showed a unique substitution N94T. N94 was retained in all isolates including Ck/India/WB-NIV529/08, Assam and Tripura, Ck/India/WB-NIV16915/08, all 2009 Indian isolates as well as all the 2007-09 Bangladesh isolates and the 2007 Kuwait and Saudi Arabia isolates. The Assam (Ck/India/AS-NIV15983/08) and Tripura (Dk/India/TR-NIV4396/08) isolates acquired substitutions R189K and I282V in the HA gene as in few of the Bangladesh 2008 isolates. I282V was also found in the HA gene of the only human isolate from Bangladesh (Bangladesh/207095/09). One additional unique mutation each, viz I83V in Assam and R162M in Tripura were also observed. The 2009 Indian isolates were seen to possess additional mutations. Of these N84D, S129L, R310K and V533M were unique while D124E and V312I were also present in Ck/India/WB-NIV529/08, Ck/India/WB-NIV16915/08 as also in 3 Bangladesh isolates of 2007-08 (Table 1, Figure 2A). Further, in the HA gene, the Ck/India/WB-NIV2812/08 isolate of Birbhum showed a maximum of six unique mutations when compared to other Indian isolates.

**Table 1:** Unique substitutions in HA gene of 2008 Indian isolates with respect to 2006 and 2007 Indian isolates.

Residue position (H5 numbering)	Ck/India/NIV33487/06	Ck/Manipur/NIV9743/07	Ck/India/WB-NIV527/08	Ck/India/WB-NIV529/08	Ck/India/WB-NIV2653/08	Ck/India/WB-NIV2654/08	Ck/India/WB-NIV2656/08	Ck/India/WB-NIV2664/08	Ck/India/WB-NIV2665/08	Ck/India/WB-NIV2670/08	Ck/India/WB-NIV2800/08	Ck/India/WB-NIV2805/08	Ck/India/WB-NIV2806/08	Ck/India/WB-NIV2807/08	Ck/India/WB-NIV2811/08	Ck/India/WB-NIV2812/08	Ck/India/WB-NIV2813/08	Dk/India/TR-NIV4396/08	Ck/India/AS-NIV15983/08	Ck/India/WB-NIV16915/08	Ck/India/WB-NIV16924/09	Ck/India/WB-NIV2456/09	Ck/India/WB-NIV6526/09	Ck/Bangladesh/362/07	Ck/Bangladesh/364/07	Ck/Bangladesh/380/07	Ck/Bangladesh/382/07	Ck/Bangladesh/394/07	Ck/Bangladesh/490/07	Ck/Bangladesh/531/07	Ck/Bangladesh/FDILG514/07	Ck/Bangladesh/FDILJ32/07	Ck/Bangladesh/CD-48-BL-165/08	Ck/Bangladesh/CD586-08-BL-409/08	Ck/Bangladesh/FDB-120-BL-148/08	Ck/Bangladesh/FDG-170-BL-141/08	Ck/Bangladesh/FDG-91-BL-415/08	Ck/Bangladesh/FDJ-2550BL-411/08	Ck/Bangladesh/FDJ-424-BL-152/08	Ck/Bangladesh/FDJ-435-BL-156/08	Ck/Bangladesh/FDJ-462-BL-195/08	Ck/Bangladesh/FDS-44-BL-144/08	Bangladesh/207095/08	Ck/Bangladesh/CD-0809BL-418/09	HB/SaudiArabia/6732-1/07	Ck/Kuwait/KISR2/07
15	Q	.	.	.	.	.	.	.	.	.	R	.	.	.	.	.	.	.	.	.	.	.	.	.	.	.	.	.	.	.	.	.	.	.	.	.	.	.	.	.	.	.	.	.	.	.
83	I	.	.	.	.	.	.	.	.	.	.	.	.	.	.	.	.	.	V	.	.	.	.	.	.	.	.	.	.	.	.	.	.	.	.	.	.	.	.	.	.	.	.	.	.	.
84	N	.	.	.	.	.	.	.	.	.	.	.	.	.	.	.	.	.	.	.	D	D	D	.	.	.	.	.	.	.	.	.	.	.	.	.	.	.	.	.	.	.	.	.	.	.
88	D	.	.	.	.	.	.	.	.	.	.	.	.	.	.	G	.	.	.	.	.	.	.	.	.	.	.	.	.	.	.	.	.	.	.	.	.	.	.	.	.	.	.	.	.	.
94	N	.	T	.	T	T	T	T	T	T	T	T	T	T	T	T	T	.	.	.	.	.	.	.	.	.	.	.	.	.	.	.	.	.	.	.	.	.	.	.	.	.	.	.	.	.
112	E	.	.	.	.	.	.	.	.	.	.	.	.	.	.	K	.	.	.	.	.	.	.	.	.	.	.	.	.	.	.	.	.	.	.	.	.	.	.	.	.	.	.	.	.	.
124	D	.	.	E	.	.	.	.	.	.	.	.	.	.	.	.	.	.	.	E	E	E	E	.	.	.	.	.	.	.	.	E	.	.	.	.	.	.	.	E	E	.	.	.	.	.
129	S	.	.	.	.	.	.	.	.	.	.	.	.	.	.	.	.	.	.	.	L	L	L	.	.	.	.	.	.	.	.	.	.	.	.	.	.	.	.	.	.	.	.	.	.	.
155	D	N	.	.	N	N	G	N	.	.	.	.	N	.	.	.	.	.	.	.	.	.	.	.	.	.	.	.	.	G	.	.	.	.	.	.	.	.	.	.	.	.	.	.	.	.
162	R	.	I	I	I	I	I	I	I	I	I	I	I	I	I	I	I	M	I	I	I	I	I	I	I	I	I	I	I	I	I	I	I	I	I	I	.	I	I	I	I	I	I	I	I	I
189	R	.	.	.	.	.	.	.	.	.	.	.	.	.	.	.	.	K	K	.	.	.	.	.	.	.	.	.	.	.	.	.	.	K	.	.	K	K	.	.	.	.	.	K	.	.
198	I	.	.	.	.	.	.	.	.	.	.	.	.	.	.	T	.	.	.	.	.	.	.	.	.	.	.	.	.	.	.	.	.	.	.	.	.	.	.	.	.	.	.	.	.	.
207	Q	.	L	L	L	L	L	L	L	L	L	L	L	L	L	L	L	L	L	L	L	L	L	L	L	L	L	L	L	L	L	L	L	L	L	L	L	L	M	L	L	L	L	L	.	.
217	S	.	.	.	.	.	.	.	P	P	.	.	.	.	.	.	.	.	.	.	.	.	.	.	.	.	.	.	.	.	.	.	.	.	.	.	.	.	.	.	.	.	.	.	.	.
244	N	.	.	.	.	.	D	D	.	.	D	.	.	.	.	.	D	.	.	.	.	.	.	.	.	.	.	.	.	.	.	.	.	.	.	.	.	.	.	.	.	.	.	.	.	.
277	K	.	.	.	.	.	.	.	R	.	.	.	.	.	.	.	.	.	.	.	.	.	.	.	.	.	.	.	.	.	.	.	.	.	.	.	.	.	.	.	.	.	.	.	.	.
282	I	.	.	.	.	.	.	.	.	.	.	.	.	.	.	.	.	V	V	.	.	.	.	.	.	.	.	.	.	.	.	.	V	V	V	.	V	V	.	.	.	.	V	V	.	.
289	M	.	.	.	.	.	.	.	.	.	.	.	.	.	.	L	.	.	.	.	.	.	.	.	.	.	.	.	.	.	.	.	.	.	.	.	.	.	.	.	.	.	.	.	.	.
309	N	.	.	.	T	T	.	.	.	.	.	.	T	.	.	.	.	.	.	.	.	.	.	.	.	.	.	.	.	.	.	.	.	.	.	.	.	.	.	.	.	.	.	.	.	.
310	R	.	.	.	.	.	.	.	.	.	.	.	.	.	.	.	.	.	.	.	K	K	K	.	.	.	.	.	.	.	.	.	.	.	.	.	.	.	.	.	.	.	.	.	.	.
312	V	.	.	I	.	.	.	.	.	.	.	.	.	.	.	.	.	.	.	I	I	I	I	.	.	.	.	.	.	.	.	I	.	.	.	.	.	.	.	I	I	.	.	.	.	.
321	P	.	.	.	.	.	.	.	.	.	.	.	.	.	.	H	.	.	.	.	.	.	.	.	.	.	.	.	.	.	.	.	.	.	.	.	.	.	.	.	.	.	.	.	.	.
355	H	.	.	.	.	.	.	.	.	.	.	.	.	.	.	.	.	.	.	.	.	.	L	.	.	.	.	.	.	.	.	.	.	.	.	.	.	.	.	.	.	.	.	.	.	.
396	V	.	.	.	.	.	.	.	.	.	.	.	.	.	.	I	.	.	.	.	.	.	.	.	.	.	.	.	.	.	.	.	.	.	.	.	.	.	.	.	.	.	.	.	.	.
457	R	.	.	.	.	.	.	.	.	.	.	.	M	.	.	.	.	.	.	.	.	.	.	.	.	.	.	.	.	.	.	.	.	.	.	.	.	.	.	.	.	.	.	.	.	.
464	G	.	.	V	.	.	.	.	.	.	.	.	.	.	.	.	.	.	.	.	.	.	.	.	.	.	.	.	.	.	.	.	.	.	.	.	.	.	.	.	.	.	.	.	.	.
482	V	.	.	.	.	.	.	.	.	.	.	.	.	.	.	.	.	.	.	I	.	.	.	.	.	.	.	.	.	.	.	.	.	.	.	.	.	.	.	.	.	.	.	.	.	.
496	A	.	S	S	S	S	S	S	S	S	S	S	S	S	S	S	S	S	S	S	S	S	S	S	S	S	S	S	S	S	S	S	S	S	S	S	S	S	S	S	S	S	S	S	S	S
533	V	.	.	.	.	.	.	.	.	.	.	.	.	.	.	.	.	.	.	.	M	M	M	.	.	.	.	.	.	.	.	.	.	.	.	.	.	.	.	.	.	.	.	.	.	.

**Table 2:** Unique substitutions in NA gene of 2008 Indian isolates with respect to 2006 and 2007 Indian isolates.

Residue position (N1 numbering)	Ck/India/NIV33487/06	Ck/Manipur/NIV9743/07	Ck/India/WB-NIV527/08	Ck/India/WB-NIV529/08	Ck/India/WB-NIV2653/08	Ck/India/WB-NIV2654/08	Ck/India/WB-NIV2656/08	Ck/India/WB-NIV2664/08	Ck/India/WB-NIV2665/08	Ck/India/WB-NIV2670/08	Ck/India/WB-NIV2800/08	Ck/India/WB-NIV2805/08	Ck/India/WB-NIV2806/08	Ck/India/WB-NIV2807/08	Ck/India/WB-NIV2811/08	Ck/India/WB-NIV2812/08	Ck/India/WB-NIV2813/08	Dk/India/TR-NIV4396/08	Ck/India/AS-NIV15983/08	Ck/India/WB-NIV16915/08	Ck/India/WB-NIV16924/09	Ck/India/WB-NIV2456/09	Ck/India/WB-NIV6526/09	Bangladesh/207095/08	HB/SaudiArabia/67321/07	Ck/Kuwait/KISR2/07
16	V	.	A	.	.	.	.	A	A	.	.	A	.	A	A	.	.	.	.	.	.	.	.	.	.	.
29	M	.	.	.	.	.	.	.	.	.	.	.	.	.	.	.	.	I	.	.	V	V	V	.	.	.
31	S	.	.	.	.	.	.	.	.	.	.	P	.	.	.	.	.	.	.	.	.	.	.	.	.	.
32	I	.	V	.	V	V	V	V	V	V	V	V	V	V	V	V	V	.	.	.	.	.	.	.	.	.
38	I	.	.	.	.	.	.	.	.	.	.	.	.	.	.	.	.	.	.	.	V	V	V	.	L	.
39	Q	.	.	.	.	.	.	.	.	.	.	.	.	.	.	.	.	.	H	.	.	-	.	.	L	L
40	T	.	.	.	.	.	.	.	.	.	.	.	.	.	.	.	.	.	.	A	.	.	.	.	.	.
41	G	.	R	M	R	R	R	R	R	R	R	R	R	R	R	R	R	R	R	M	M	M	M	R	R	R
45	Q	.	.	.	.	.	P	.	.	.	.	.	.	.	.	.	.	.	.	.	.	.	.	.	.	.
69	I	.	.	.	.	.	.	.	.	.	.	.	.	.	.	S	.	.	.	.	.	.	.	.	.	.
71	N	.	.	.	.	.	.	.	.	.	.	.	.	.	.	.	.	.	.	.	S	S	S	.	.	.
72	T	A	.	.	.	.	.	.	.	.	.	.	.	.	.	.	.	.	.	.	S	S	S	.	.	.
74	F	.	.	L	.	.	.	.	.	.	.	.	.	.	.	.	.	.	.	L	.	.	.	.	.	.
75	L	.	.	.	.	.	.	.	.	.	.	.	.	.	.	.	.	.	.	.	H	H	H	.	.	.
76	T	.	.	.	.	.	.	.	.	.	.	.	.	.	.	.	.	.	.	.	.	.	A	.	.	.
83	V	.	.	.	.	.	.	.	.	L	.	.	.	.	.	.	.	.	.	.	.	.	.	.	.	.
86	A	.	.	.	.	.	.	.	.	P	.	.	.	.	.	.	.	.	.	.	.	.	.	.	.	.
95	S	.	.	.	.	.	.	.	.	N	.	.	.	.	.	.	.	.	.	.	.	.	.	.	.	.
102	K	.	.	.	.	.	.	.	.	R	.	.	.	.	.	.	.	.	.	.	.	.	.	.	.	.
117	I	.	.	.	.	.	.	.	.	.	.	.	.	.	.	.	.	.	.	V	.	.	.	.	.	.
119	E	.	.	A	.	.	.	.	.	.	.	.	.	.	.	.	.	.	.	A	.	.	.	.	.	.
126	H	.	.	Y	.	.	.	.	.	.	.	.	.	.	.	.	.	.	.	Y	.	.	.	.	.	.
155	H	.	.	.	.	.	.	.	.	.	.	.	.	.	.	.	.	.	.	.	Q	Q	Q	.	.	.
165	E	.	.	.	.	.	.	.	.	.	.	.	.	.	.	.	.	.	.	.	.	K	.	.	.	.
196	S	.	.	.	.	.	.	.	.	.	.	Y	.	.	.	.	.	.	.	.	.	.	.	.	.	.
198	P	.	.	.	Q	.	.	.	.	.	.	.	.	.	.	.	.	.	.	.	.	.	.	.	.	.
205	V	.	.	.	.	.	.	.	.	.	.	.	.	I	.	.	.	.	.	.	.	.	.	.	.	.
221	N	.	.	.	.	.	D	.	.	.	.	.	.	.	.	.	.	.	.	.	.	.	.	.	.	.
241	V	.	.	.	.	.	.	.	.	.	.	.	.	.	.	.	.	.	.	.	.	.	I	.	.	.
249	G	.	.	.	.	.	.	.	.	E	.	.	.	.	.	.	E	.	.	.	.	.	.	.	.	.
254	K	.	.	.	.	.	.	.	.	.	.	.	.	M	.	.	.	.	.	.	.	.	.	.	.	.
258	M	.	.	.	.	.	.	.	.	.	.	.	.	.	.	.	.	.	.	.	.	.	I	.	.	.
264	V	I	.	.	.	.	I	.	.	.	.	.	.	.	.	.	.	.	.	.	.	.	.	.	.	.
267	I	V	V	V	V	V	V	V	V	V	V	V	V	V	V	V	V	V	V	V	V	.	V	V	V	V
285	A	T	.	.	.	.	.	.	.	.	.	.	.	.	.	.	.	.	.	.	.	.	.	.	.	.
286	G	.	.	.	.	.	.	.	.	.	.	.	.	.	.	.	.	.	.	S	.	.	.	.	.	.
295	N	.	.	.	.	.	.	S	S	.	.	.	.	.	.	.	.	.	.	.	.	.	.	.	.	.
311	E	.	.	D	.	.	.	.	.	.	.	.	.	.	.	.	.	.	.	D	.	.	.	.	.	.
340	P	S	L	L	L	.	L	L	L	L	L	L	L	L	L	L	L	L	L	L	L	L	L	L	L	L
376	D	.	.	.	.	.	.	.	.	.	.	.	.	.	E	.	.	.	.	.	.	.	.	.	.	.
381	T	.	.	.	.	.	.	.	.	.	.	.	.	.	.	.	.	.	.	.	A	A	A	.	.	.
383	T	.	.	.	.	.	.	.	.	.	.	.	.	.	.	.	.	.	A	.	.	.	.	.	.	.
386	S	.	.	.	.	.	.	.	.	.	.	.	.	.	.	.	.	R	H	.	.	.	.	.	.	.
430	R	.	Q	Q	Q	Q	.	Q	Q	Q	Q	Q	Q	Q	.	Q	Q	Q	Q	Q	Q	Q	Q	Q	.	.
449	N	.	.	.	.	.	.	.	.	.	.	.	.	.	.	.	.	S	S	.	.	.	.	.	.	.

In the NA gene (Table 2), substitutions I267V and P340L in majority of the Indian isolates were as in the 2007 isolates of Kuwait, Saudi Arabia and the only available human isolate from Bangladesh (Bangladesh/207095/09). G41R substitution in NA of all 2008 Indian isolates (except Ck/India/WB-NIV529/08, Ck/India/WB-NIV16915/08 and all 2009 Indian isolates) was as in the 2007 isolates of Kuwait, Saudi Arabia and Bangladesh/207095/09. All 2009 Indian isolates including Ck/India/WB-NIV529/08 and Ck/India/WB-NIV16915/08 showed G41M mutation. Further, all early 2008 Indian isolates (except Ck/India/WB-NIV529/08) showed a unique mutation I32V in the NA gene. Additionally, R430Q was seen in majority of the Indian 2008-09 isolates as also in Bangladesh/207095/09. The Ck/India/WB-NIV529/08 isolate of South Dinajpur, WB and the late 2008 isolate Ck/India/WB-NIV16915/08 of Malda, WB shared 4 mutations viz. F74L, E119A, H126Y and E311D. Further, Assam and Tripura isolates shared 32I with the WB 2008 isolates from South Dinajpur (Ck/India/WB-NIV529/08), Malda (Ck/India/WB-NIV16915/08) as well as the 2009 Indian isolates as in the human isolate from Bangladesh 2007 and isolates of Kuwait, Saudi Arabia and Krasnodar. All the 2009 Indian isolates possessed additional unique substitutions M29V, I38V, N71S, T72S, L75H, H155Q and T381A indicative of further diversification.

### Molecular Characterization

All of the 2008-09 Indian isolates possess the polybasic endo-proteolytic cleavage site GERRRKKR in the HA gene [13]–[14] prominent for highly pathogenic avian influenza A viruses. Though majority of WB isolates as well as Tripura and Assam isolates have S155D (reference as Vietnam/1203/04) in HA1, few isolates of WB (Ck/India/WB-NIV2664/08 of Birbhum, Ck/India/WB-NIV2806/08 of Bardhaman, Ck/India/WB-NIV2653/08 of Nadia and Ck/India/WB-NIV2654/08 of Malda have N, while Ck/India/WB-NIV2656/08 of Murshidabad has G at position 155. This is the known antigenic site B [15] and hence may have a role to play in overcoming host immune responses.

Several residues, in particular 221 and 223 (226 and 228 respectively in H3 numbering) in the receptor binding domain (RBD) of the HA1 protein have been linked to receptor specificity [16]. Based on structural comparisons of the RBDs, it was shown that mutations Q221L and G223S enable H3 viruses to become human adapted. So also a mutation E186D (190 in H3 numbering), affects the width of the binding site and favours accommodation of human alpha 2, 6 linked sugars in H5 viruses. All 2008-09 Indian viruses and also the earlier H5N1 Indian viruses of 2006 and 2007 and most of clade 2.2 viruses have retained Q221, G223 and E186. Among other critical substitutions implicated in receptor specificity that are based on glycan microarray analysis of H1 and H5 viruses [17] a mutation R212K (216 in H3 numbering,) has been observed in all the Indian isolates while K189R (193 in H3 numbering) has been observed in all Indian isolates except the Assam and Tripura isolates. Two isolates from Birbhum district of WB (Ck/India/WB-NIV2665/08, Ck/India/WB-NIV2670/08) showed S217P (221 in H3 numbering) mutation (Table 1).

A substitution E119A was observed in Ck/India/WB-NIV529/08 isolate of South Dinajpur, WB and the late 2008 Malda isolate, Ck/India/WB-NIV16915/08 while N295S (294 in N2 numbering) was observed in two isolates Ck/India/WB-NIV2664/08 and Ck/India/WB-NIV2665/08 of Birbhum, WB (Table 2). These substitutions in the active site of neuraminidase [18] are known to confer Oseltamivir and Zanamivir resistance [19], [20]. The 2008-09 Indian isolates showed sensitivity to Amantadine on the basis of known markers in the M2 ion channel protein [21]. There are 32 amino acid residues in PB2, PA, NP, M1 and M2 proteins that had been described as host specific amino acid residues [22]. Of these, all the amino acid residues at these sites in our isolates suggest avian specificity except V28 in the M2 protein and V33I in the NP protein [22]. In addition, in the PB2 gene, a substitution E627K was described to be associated with virulence in mice and adaptation to humans [23]. The E627K substitution was found in all the 2008-09 Indian isolates (as in several other clade 2.2 isolates) except the Tripura isolate. Of the known markers for increased polymerase activity [24] the substitutions, L13P and N678S in PB1 protein are observed in the 2008-09 Indian isolates. 92E of NS1, a high virulence indicator that helps to overcome host innate immune responses [25] was observed in the 2008-09 Indian isolates. The deviations in the marker residues stated above or the observance of specific markers have been found to occur in other clade 2.2 viruses. The terminal four amino acids of NS1, known as a functional PDZ-binding domain [26] was found to be ESKV in all 2008-09 Indian isolates as well as the earlier Indian isolates. Unlike the 2007 isolate from Manipur [9] all the 2008-09 Indian isolates possessed full length PB1 F2 protein. Analysis of the PB1 F2 protein further showed that one of the WB isolates possessed a difference of four amino acids (data not shown) compared to the other 2008-09 Indian isolates as well as the earlier Indian isolates, the significance of which is yet to be elucidated.

## Discussion

The phylogenetic analysis of the Indian isolates and other global isolates showed clustering of the 2008-09 Indian isolates in all the eight genes with the isolates of 2007 from Kuwait, Saudi Arabia, Krasnodar, Germany, Romania and the Czech Republic. According to the recent WHO H5N1 nomenclature system [27] these viruses fall within a possible third order clade of clade 2.2. The relationship of the Indian 2008-09 isolates is indicative of their ancestral relationship with strains from Middle East, Russia and Europe. Lesser similarity with the earlier Indian isolates of 2006 and 2007 implies that the 2008-09 outbreaks may not have resulted from local evolution and may be considered as an independent re-introduction into the country. The Kuwait outbreaks that were first reported in February 2007 were followed by outbreaks in several countries including Myanmar in February 2007, Bangladesh and Saudi Arabia around March-April 2007 [28], and India in July 2007 [9] followed by the 2008-09 outbreaks. Of these the Myanmar isolates (Ck/Hmawbi/517/07 and Quail/Mingalardone/866/07) were found to be phylogenetically distinct, belonging to clade 2.3 in the HA gene (Figure. 2A) and hence the possible introduction of the Myanmar viruses in the perspective of the 2008-09 outbreaks in India can be ignored. Avian influenza outbreaks in Bangladesh have been continuing since March 2007 [28], [29]. A first phase of H5N1 in Bangladesh was observed till October 2007 followed by a second phase (phase II) between December 2007 and April 2008, wherein 156 outbreaks were reported from 6 provinces. Subsequently, outbreaks of H5N1 infection were reported in Bangladesh from November 2008 onwards (phase III). The periods of the phase II and III outbreaks of Bangladesh coincided with phase I (January–May 2008) and phase II (November 2008 onwards) H5N1 outbreaks in India. Earlier reports [29] have shown that the Bangladesh isolate belong to clade 2.2 of the Qinghai lineage and are most closely related to viruses from Afghanistan, Mongolia and Russia [30]. Our phylogenetic analysis indicated the close relatedness of the 2008-09 Indian isolates with the 2007-09 Bangladesh isolates. In the HA gene phylogenetic tree, the Indian isolates of 2008-09 clustered along with Bangladesh isolates. Within this cluster the 2007 isolates of Bangladesh shared closer identity with the 2007 isolates of Kuwait and Saudi Arabia. This may be indicative that the Kuwait and Saudi Arabia viruses are the possible ancestral source of the viruses in Bangladesh. Notably, from the timeline it is clear that, the outbreaks reported in Bangladesh coincide with those of Saudi Arabia, which were preceded by the Kuwait outbreaks. Whether migratory birds or trade between these countries would be the possible cause of these outbreaks is yet to be understood.

With the first wave of the outbreak in January 2008 in India, almost the whole of southern WB was affected (Figure 1). During March–May 2008 (second wave of the outbreak), the infection spread to the northern districts of WB, viz Darjeeling and Jalpaiguri, and also to a major part of Tripura along with repeated outbreaks in few of the earlier affected districts of southern WB. In the third wave of the outbreak (November 2008–May 2009), the movement of the infection was towards the northeast with several districts of Assam and the northern parts of WB being affected.

Bayesian analysis of the phylogenetic relationships among the 2008-09 Indian isolates and 2007-09 Bangladesh isolates (Figure 3) indicated that the cluster of Indian and Bangladesh isolates could be further divided into three main subgroups. Interestingly, in each subgroup, the Indian isolates formed separate clusters more or less independent of the Bangladesh isolates of 2007-09. All but the South Dinajpur isolate of the early phase I outbreak (January 2008) clustered together in the first subgroup, while only the South Dinajpur (Ck/India/WB-NIV529/08) and the late 2008 isolate of Malda (Ck/India/WB-NIV16915/08) clustered into the second subgroup along with other 2007-08 isolates of Bangladesh. Based on the observations it appears that there have been multiple sources of the virus into the country during the early phase I outbreaks in India. The viruses of Tripura (late phase I outbreaks) and Assam (phase II) shared relatedness with another isolate of Bangladesh (Ck/Bangladesh/BL411/08) and so also the 2009 isolates of India were found to be related with other Bangladesh isolates, implying either other sources of the virus into these states or in-situ evolution and spread within the country. However, the transmission of viruses between the two countries in either direction cannot be denied. Altogether, it appears that the region including Bangladesh and the East and Northeast parts of India have become endemic to the H5N1 virus.

The whole genome sequencing of the four isolates selected from the different clusters discussed above, represent different time points of infection and independent outbreak incidents. Further, the mutations observed in the isolates from WB, Tripura and Assam (Table 1, 2) is an indication of genetic diversification. The molecular characterization in terms of pathogenic markers indicated that all 2008-09 Indian isolates were highly virulent in chickens. Though the majority of markers at the receptor binding site of HA was suggestive of avian receptor specificity, the S221P mutation in the HA gene of two WB isolates of the present study may imply possible specificity to human receptor [17]. It has been reported that a double mutation E216R/K, P221S that indicates avian receptor alpha 2–3 linkage specificity was observed in all Z-genotype isolates from 2003 onwards [17]. Interestingly, only a few strains from the Z genotype show S221P mutation with the 216 R/K. To the best of our knowledge, as compared with the available sequences in public database, our two WB isolates are the first representatives in clade 2.2 that have the S221P mutation, with the K216 being retained. Notably, the double substitution, R216E and S221P in the A/Hong Kong/156/97 isolate has been shown to decrease binding to branched fucosylated glycans [31] and favour α2–6 linkages in human receptors. Further study is therefore important to understand the significance of the S221P mutation in the two WB virus isolates. Though the mutation may be a step towards gaining a human foothold, no human cases have been reported during the entire duration of the 2008-09 Indian outbreaks. In-fact, none of the Indian outbreaks since 2006 have been associated with human infection. The only reported human infection in the region [12] is reflected in the single isolate of Bangladesh (A/Bangladesh/207095/08), which did not show the above-mentioned mutations. However, the overall similarity of the human isolate from Bangladesh with the Indian isolates of 2008-09, specifically the isolates from Assam and Tripura, is significant as a potential alert for possible transmission into humans.

Several studies [32], [33] have indicated the mutations in the NA gene that confer resistance to known NA inhibitors such as Oseltamivir and Zanamivir. Reduced sensitivity to Oseltamivir has been reported in some isolates belonging to different clades, including clade 1 Cambodian isolates and clade 2 Indonesian isolates from 2005 [34], clade 2.3.4 and clade 3 [35]. Because none of the observed sequence variations correlated with the well known mutations known to confer Oseltamivir resistance and none of the variations were in the active site [10], it was suggested that the decrease in sensitivity might be due to drift mutations rather than from exposure to Oseltamivir. On the other hand, Oseltamivir-resistant H5N1 viruses with amino acid changes at the NA active site such as H274Y and N294S have emerged in humans during Oseltamivir treatment [36]–[38].

Recently [39], two human isolates from Egypt were seen to possess N294S with no history of Oseltamivir treatment. Other studies showed that human isolates could also demonstrate decreased sensitivity to Oseltamivir and Zanamivir with drift mutations in the NA, remote from the active site [40]. In this study, we observed a mutation E119A in the NA gene of two of the WB isolates and N294S in two other isolates. While the mutation at position N294S is a well characterized neuraminidase inhibitor resistance marker [20], the mutation at E119A is not as well studied in the context of H5N1 viruses, though it is known to be a key residue in the active site of NA [18]. A recent study [19], based on NA enzyme inhibition assays using reverse genetics modified recombinant viruses (A/Turkey/15/06-like H5N1), has shown that most of the known NA mutations including E119A conferred resistance to Oseltamivir whereas resistance to Zanamivir was found only with substitutions at V116A and E119A. On comparison of the existing NA sequences of H5N1 viruses in the GenBank, E119A was not observed, while N294S was seen in only a few isolates (2 human isolates from Egypt, 2006 and 2 duck isolates from China, 2001–2004). No antiviral resistance assays have been performed in our laboratory to provide additional evidence that these mutations confer resistance in the WB virus. However, in view of the above discussion, the mutations observed in the NA of the two Indian isolates is noteworthy and raises concern towards effectiveness of the antivirals in prophylactic measures that are presently being adopted in the country.

Overall, molecular and phylogenetic characterization of Influenza A H5N1 viruses of the 2008-09 outbreaks in India suggest at least a third independent introduction into the country. Though migratory birds may have a role in the possible transmission of H5N1 viruses, the possibility via illegal poultry trade from neighbouring countries cannot be denied. Finally, our study has shown that the virus is endemic in the region including east and north eastern parts of India as well as the neighbouring country Bangladesh and demands active surveillance and concentrated efforts to control the spread specifically in view of the critical mutations that have been observed in this virus.

## Materials and Methods

### Virus Isolation and Identification

Clinical specimens were received from sick/dead birds, from the affected areas. Lung, trachea, liver, kidney, spleen and brain tissue samples, cloacal swabs as well as faecal specimens were processed in viral media and directly used for virus isolation using Specific-pathogen-free (SPF) embryonated White Leghorn chicken eggs and Madin Darby Canine Kidney (MDCK) cell lines [8], [9]. Allantoic fluid was harvested after observing inoculated eggs for 48 hours. All the samples were processed in the enhanced Biosafety level three (BSL-3+) laboratory. Hemagglutination (HA) and Hemagglutination Inhibition (HAI) tests were performed using horse and fowl RBC as described earlier [41], [42].

### RNA Extraction and Genome Sequencing

Viral RNA was isolated from the clinical specimens and allantoic fluid/tissue culture supernatant using QIAamp viral mini kit (Qiagen, Germany). One step RT PCR and real time RT-PCR tests [42] were performed as mentioned earlier [9]. cDNA prepared from extracted viral RNA using Uni 12 primer was used to amplify all the eight viral gene segments using segment specific primers [43]. Roche HiFi PCR system (Roche, Germany) was used for all the PCR amplifications. The PCR products were separated in an agarose gel by electrophoresis; amplicons of the appropriate sizes were subsequently excised from the gel and purified using QIAGEN gel extraction kit (Qiagen, Germany). Purified PCR products were directly used for cycle sequencing reactions. Sequencing of the amplified products was done on an automated Applied Biosystems' 3130 XL system using cycle sequencing big dye terminator (ABI). Table S1 provides the details of the isolates, their collection date, place of isolation, passage history, the genes sequenced and their GenBank accession numbers.

### Phylogenetic Analysis

Representative sequences of the H5N1 viruses belonging to the Z genotype were selected from the GenBank based on sequence identity (100% identical sequences were excluded) and including wherever possible isolates whose whole genomes were available. Multiple nucleotide and amino acid sequence alignments for all eight gene segments were performed using Clustalx version 1.83 [44]. Phylogenetic analysis was performed using the neighbour-joining method with the Kimura 2-parameter distance model and 1000 bootstrap replicates using MEGA version 4 [45]. The percent nucleotide identity (PNI) and percent amino acid identity (PAI) values were calculated as pairwise p-distances. The tree topologies were confirmed by using the Bayesian approach for tree construction as implemented in Mr Bayes 3.2 [46]. The GTR (General Time Reversible)+I (Invariable sites) model with gamma-distributed rate variation across sites and a proportion of invariable sites, was specifically used and other parameters were kept as default.

## Supporting Information

Table S1 Details of viruses isolated and analysed during January 2008 - May 2009.
(0.04 MB DOC)
