# Supplementary material for: Characterization of the Influenza A H5N1 Viruses of the 2008-09 Outbreaks in India Reveals a Third Introduction and Possible Endemicity
Source: PLoS One. 2009 Nov 16;4(11):e7846. doi: 10.1371/journal.pone.0007846 (PMC2775943; doi:10.1371/journal.pone.0007846)
Supplement: Table S1 — Details of viruses isolated and analysed during January 2008 - May 2009. (0.04 MB DOC) [file pone.0007846.s001.doc]

| Sl. No. | Name of the isolate | Date of Isolation | Source | Place of collection | Passage History | Gene Sequenced | GenBank Accession Number |
| --- | --- | --- | --- | --- | --- | --- | --- |
| 1 | A/Chicken/India/WB- NIV527/2008 | 13-Jan-2008 | Chicken | Birbhum, WB | Passage 1 | HA and NA | CY046107, CY046108 |
| 2 | A/Chicken/India/WB- NIV529 /2008 | 13-Jan-2008 | Chicken | South Dinajpur, WB | Passage 1 | PB2, PB1, PA, HA, NP, NA, M, NS | CY046109 -CY046116 |
| 3 | A/Chicken/India/WB-NIV2653/2008 | 21-Jan-2008 | Chicken | DeyPara, Nadia, WB | Passage 1 | PB2, PB1, PA, HA, NP, NA, M, NS | CY046067-CY046074 |
| 4 | A/Chicken/India/WB-NIV2654/2008 | 21-Jan-2008 | Chicken | Mahanandapur, Malda, WB | Passage 1 | HA and NA | CY046075-CY046076 |
| 5 | A/Chicken/India/WB-NIV2656/2008 | 21-Jan-2008 | Chicken | Bharatpur, Murshidabad, WB | Passage 1 | HA and NA | CY046077- CY046078 |
| 6 | A/Chicken/India/WB-NIV2664/2008 | 22-Jan-2008 | Chicken | Akuni, Birbhum, WB | Passage 1 | HA and NA | CY046079- CY046080 |
| 7 | A/Chicken/India/WB-NIV2665/2008 | 22-Jan-2008 | Chicken | Thanapara, Birbhum, WB | Passage 1 | HA and NA | CY046081- CY046082 |
| 8 | A/Chicken/India/WB-NIV2670/2008 | 22-Jan-2008 | Chicken | Rajarpukur, Birbhum, WB | Passage 1 | HA and NA | CY046083-CY046084 |
| 9 | A/Chicken/India/WB-NIV2800/2008 | 23-Jan-2008 | Chicken | Daspara, Nadia | Passage 1 | HA and NA | CY046085-CY046086 |
| 10 | A/Chicken/India/WB-NIV2805/2008 | 23-Jan-2008 | Chicken | Majiara, Bardhaman, WB | Passage 1 | HA and NA | CY046087-CY046088 |
| 11 | A/Chicken/India/WB-NIV2806/2008 | 23-Jan-2008 | Chicken | Domohani, Bardhaman, WB | Passage 1 | HA and NA | CY046089-CY046090 |
| 12 | A/Chicken/India/WB-NIV2807/2008 | 23-1-2008 | Chicken | Karanbad, Bardhaman, WB | Passage 1 | HA and NA | CY046091-CY046092 |
| 13 | A/Chicken/India/WB-NIV2811/2008 | 25-Jan-2008 | Chicken | Siulia, Birbhum, WB | Passage 1 | HA and NA | CY046093-CY046094 |
| 14 | A/Chicken/India/WB-NIV2812/2008 | 25-Jan-2008 | Chicken | Suchpur, Birbhum, WB | Passage 1 | HA and NA | CY046095-CY046096 |
| 15 | A/Chicken/India/WB-NIV2813/2008 | 25-Jan-2008 | Chicken | Bhatier, Birbhum, WB | Passage 1 | HA and NA | CY046097- CY046098 |
| 16 | A/Duck/India/TR-NIV4396/2008 | 03-Apr-2008 | Duck | Agartala, Tripura | Passage 1 | PB2, PB1, PA, HA, NP, NA, M, NS | CY046099- CY046107 |
| 17 | A/Chicken/India/AS-NIV15983/2008 | 27-Nov-2008 | Chicken | Kamrup, Assam | Passage 1 | HA and NA | GQ917223- GQ917224 |
| 18 | A/Chicken/India/WB-NIV16915/2008 | 16-Dec-2008 | Chicken | Malda, WB | Passage 1 | HA and NA | GQ917225- GQ917226 |
| 19 | A/Chicken/India/WB-NIV16924/2009 | 02-Jan-2009 | Chicken | Binay Krishna pally, Darjeeling, WB | Passage 1 | HA and NA | GQ917227- GQ917228 |
| 20 | A/Chicken/India/WB-NIV2456/2009 | 10-Mar-2009 | Chicken | Mirik, Darjeeling, WB | Passage 1 | HA, NA, PB2, PB1 PA, NP, M, NS | GQ917231- GQ917238 |
| 21 | A/Chicken/India/WB-NIV6526/2009 | 23-May-2009 | Chicken | Kantor, Uttar Dinajpur, W.B | Passage 1 | HA and NA | GQ917229- GQ917230 |

Supplementary Table 1: Details of Viruses isolated and analyzed during the study period
